# Role of Glycolipids in the Pathogenesis of *Enterococcus faecalis* Urinary Tract Infection

**DOI:** 10.1371/journal.pone.0096295

**Published:** 2014-05-07

**Authors:** Ann-Kristin Diederich, Dominique Wobser, Meike Spiess, Irina G. Sava, Johannes Huebner, Türkân Sakιnç

**Affiliations:** 1 Division of Infectious Diseases, University Hospital Freiburg, Freiburg, Germany; 2 Nutrition and Immunology, Research Centre for Nutrition and Food Science, Technical University Munich, Munich, Germany; 3 Division of Pediatric Infectious Diseases, Dr. Von Hauner Children's Hospital, Ludwig Maximilian University Munich, Munich, Germany; University Medical Center Utrecht, Netherlands

## Abstract

**Background:**

After uropathogenic *Escherichia coli* (UPEC), *Enterococcus faecalis* is the second most common pathogen causing urinary tract infections. Monoglucosyl-diacylglycerol (MGlcDAG) and diglucosyl-diacylglycerol (DGlcDAG) are the main glycolipids of the *E. faecalis* cell membrane. Examination of two mutants in genes *bgsB* and *bgsA* (both glycosyltransferases) showed that these genes are involved in cell membrane glycolipid biosynthesis, and that their inactivation leads to loss of glycolipids DGlcDAG (*bgsA*) or both MGlcDAG and DGlcDAG (*bgsB*). Here we investigate the function of *bgsB* and *bgsA* regarding their role in the pathogenesis in a mouse model of urinary tract infection and in bacterial adhesion to T24 bladder epithelial cells.

**Results:**

In a mouse model of urinary tract infection, we showed that *E. faecalis* 12030Δ*bgs*B and *E. faecalis* 12030Δ*bgs*A mutants, colonize uroepithelial surfaces more efficiently than wild-type bacteria. We also demonstrated that these mutants showed a more than three-fold increased binding to human bladder carcinoma cells line T24 compared to the wild-type strain. Bacterial binding could be specifically inhibited by purified glycolipids. Lipoteichoic acid (LTA), wall-teichoic acid (WTA), and glycosaminoglycans (GAGs) were not significantly involved in binding of *E. faecalis* to the bladder epithelial cell line.

**Conclusions:**

Our data show that the deletion of *bgsB* and *bgsA* and the absence of the major glycolipid diglucosyl-diacylglycerol increases colonization and binding to uroepithelial cells. We hypothesize that secreted diglucosyl-diacylglycerol blocks host binding sites, thereby preventing bacterial adhesion. Further experiments will be needed to clarify the exact mechanism underlying the adhesion through glycolipids and their cognate receptors.

## Introduction

Urinary tract infections (UTIs) are the most common bacterial infection in the inpatient and outpatient setting, leading to about 8.6 million patient visits in the United States in 2007 [1]–[3]. The vast majority of these infections occur in women, and the costs in the USA are estimated to be over 3.5 billion dollars per year [4], [5].

The majority of UTIs are caused by gram-negative bacteria, only *Staphylococcus saprophyticus* and enterococci are clinically relevant, gram-positive pathogens causing UTIs and standard empiric therapy often does not cover these pathogens. Urinary tract infection caused by *E*. *faecalis* is the most common enterococcal infection, and after uropathogenic *Escherichia coli* (UPEC), enterococci are the second most common organisms responsible and the most common gram-positive urinary pathogen [6]. However, despite their important role in the pathophysiology of urinary tract infections, the molecular mechanisms of colonization and infection are still not well understood. In addition, *E. faecalis* is also a member of the core human gut microbiota and a leading cause of several other serious human infections, such as endocarditis [7], bacteremia [8], and wound infections [3], [9], [10], [11]. Enterococci are also found as multiresistant nosocomial pathogens, frequently related to foreign-body infections [12].

Monoglucosyl-diacylglycerol (MGlcDAG) and diglucosyl-diacylglycerol (DGlcDAG) are the main glycolipids of the *E. faecalis* cell membrane, functioning also as anchors for LTA in Gram-positive bacteria [13]. We have previously constructed and characterized two mutants (*bgsA* and *bgsB*) responsible for the production of two glycosyltransferases involved in cell membrane glycolipid biosynthesis in *E. faecalis* [13], [14]. Inactivation of *bgsB* led to the complete loss of the cell membrane glycolipids DGlcDAG and MGlcDAG, while deletion of *bgsA* abrogated addition of a second glucose molecule to MGlcDAG; either mutation alone resulted in impaired biofilm formation and adhesion to colonic epithelial cells and reduced virulence in a mouse sepsis model [13], [14]. In the present study, we further investigated the roles of *bgs*B and *bgs*A of *E. faecalis* in the pathogenesis of urinary tract infections using a cell culture model and a mouse infection model.

## Materials and Methods

### Cell culture

T24 cells derived from human bladder carcinoma (obtained from Cell Lines Service, Eppelheim, Germany) were used. Cells were cultured in Dulbecco's Modified Eagle's Medium nutrient mixture F-12 Ham (DMEM, Sigma Aldrich) supplemented with 5% fetal bovine serum (FBS, PAA The Cell Culture Company) in a humidified 5% CO2 atmosphere at 37°C as described [15].

### Bacterial strains and chemicals

*E. faecalis* 12030 is a clinical strain originally isolated in Cleveland, OH [16]. It is a strong biofilm producer and is opsonized by antibodies against lipoteichoic acid (LTA) [16]. Two non-polar deletion mutants, *E. faecalis* 12030Δ*bgs*B and *E. faecalis* 12030Δ*bgs*A together with their reconstituted strains *E. faecalis* 12030Δ*bgs*B rec and *E. faecalis* 12030Δ*bgs*A rec, have been constructed and characterized previously [13], [14]. All bacterial strains were grown at 37°C without agitation in Caso Bouillon (Carl Roth). All reagents used in this study were obtained from Sigma Aldrich if not stated otherwise.

### Adherence/Invasion assay

Cells were grown in 24-well plates to a density of 1*10^5^ cells/well for 16 hours. After 16 hours, cells were counted using trypsin treatment and trypan blue. Prior to infection of cells, bacteria were grown to mid-log phase at 37°C without agitation in Caso Bouillon (Carl Roth), washed, and resuspended in DMEM supplemented with 5% FBS. The number of bacteria in the inoculum was first estimated from previously derived growth curve determinations and confirmed by serial dilutions and viable counts for each experiment. T24 cells were incubated with bacteria at 37°C for 2 hours at a multiplicity of infection of 100∶1. After infection of the monolayers, T24 cells were washed five times with phosphate saline buffer (PBS, Biochrom AG) and lysed with DMEM F-12 Ham containing 5% FBS and 0.25% Triton-X100 buffer for 15 min. Bacteria attached and internalized by the T24 cells were quantified by cultivation of serial dilutions of the cell-culture lysates. Pilot experiments confirmed the proportion of non-adherent bacteria to be <1% of the total colony-forming units (CFUs) in the lysates. Invasion experiments were performed as described elsewhere, using the gentamycin protection assay [17].

### Inhibition of binding by glycolipids

Inhibition of bacteria adhesion to T24 cells was accomplished by the addition of partially purified glycolipids (250 µg/mL) to the cells 30 min before infection. The assay was then performed as described above. Glycolipids were isolated and purified as described by Theilacker *et al*. [13], [14].

### Inhibition of bacterial attachment by glycosaminoglycans

Glycosaminoglycans (GAGs), i.e. heparin, heparan sulfate (HS) and chondroitin sulphate B (CSB), were shown to interact with *E. faecalis* during binding of bacteria to colonic epithelial cells [18]. We tested the inhibitory effects of these compounds on adhesion of *E. faecalis* to T24 cells. In brief, GAGs were diluted in DMEM and added in various concentrations (Heparin: 10–1000 µg/mL; HS: 10–500 µg/mL; CSB: 10–1000 µg/mL) to the T24 uroepithelial cells 30 min before infection with bacteria. The binding assay was subsequently performed as described above.

### Treatment of cells with heparin lyase and chondroitin lyase ABC

To test the attachment of mutants and wild-type bacteria to T24 uroepithelial cells after depletion of surface glycosaminoglycans, heparin lyase (which cleaves heparin and HS chains) and chondroitin lyase ABC (which degrades chondroitin sulphate chains) were used as described previously [18]. In brief, heparin lyase in concentrations between 0.5 and 2 units/mL and chondroitin lyase in concentrations 0.5 and 1 units/mL were added to the T24 cells 10 min prior to infection. Additionally, 50 min after infection, the medium was supplemented with the same amount of enzyme, and the incubation continued for one additional hour.

### Sodium meta-periodate treatment

The assay was performed as previously described [18]. In brief, bacteria were incubated with various amounts of sodium meta-periodate (0.03–0.5 mM) for 60 min. at 4°C while shaking. Sodium meta-periodate was neutralized using ethylene glycol (20 µL/mL for 10 min. at room temperature on a rotor in the dark). After five washing steps with PBS buffer, bacteria were diluted 100∶1 and added to the epithelial cells as described above. Bacteria grown in the same conditions but untreated with sodium meta-periodate were used as a control.

### Proteinase K treatment

Both mutants and wild type bacteria were incubated with Proteinase K (0.1 mg/mL) for one hour at 37°C during cultivation to mid-log phase. After the incubation period, bacteria were washed five times with PBS to remove the enzyme. Bacterial viability after this treatment was confirmed by enumeration of CFUs on Caso agar. The binding assay was performed as described above.

### Mouse urinary tract infection model

Female BALB/c mice (6–8 weeks old) were used for the experiments. For the pre-culture, bacteria were grown in 5 mL of the above-described medium for ∼16 h at 37°C with gentle shaking. For inoculum preparation, the overnight culture was diluted 1∶100 in 50 mL fresh medium and grown to an optical density of 0.3 at 37°C with shaking. Cells were pelleted and resuspended in half of the volume of 0.9% saline. Serial dilutions were prepared in Caso Bouillon and then plated to determine the inoculum before injection. Bladders of isoflurane-anesthetized mice were emptied using a catheter (20 mm) before injecting bacteria. Animals were infected via urethral catheterization with 100 µL of the bacterial suspension (10^7^–10^8^ CFU per mL). After two hours, the mice were inoculated again. After the urethral catheter was removed, the animals had free access to food and water. Mice were euthanized by CO_2_ inhalation 24 h and 48 h after transurethral challenge. The urinary bladder and both kidneys were removed, weighed, and homogenized in 1 mL of 0.9% saline +0.025% Triton X-100. Dilutions were plated onto Caso Agar, and colonies were counted after overnight incubation at 37C°. The CFU/100 mg of tissue of each animal (bladder or kidney) were enumerated and groups were compared by Mann-Whitney U test.

### Statistical methods

If not stated otherwise, the experiments were repeated three times. Graph Pad Prism, version 4.0 for Windows (GraphPad Software) was used. Multigroup comparisons were made by ANOVA. P value of less than 0.05 was considered statistically significant. Error bars represent standard error of the mean.

### Ethics Statement

All animal experiments were performed in compliance with the German animal protection law (TierSchG). The mice were housed and handled in accordance with good animal practice as defined by FELASA and the national animal welfare body GV-SOLAS. The animal welfare committees of the University of Freiburg (Regierungspräsidium Freiburg Az 35/9185.81/G-11/118) approved all animal experiments.

## Results

### Deletion of genes involved in membrane glycolipid synthesis increases binding to the uro-epithelial cell line T24

**Figure 1:**
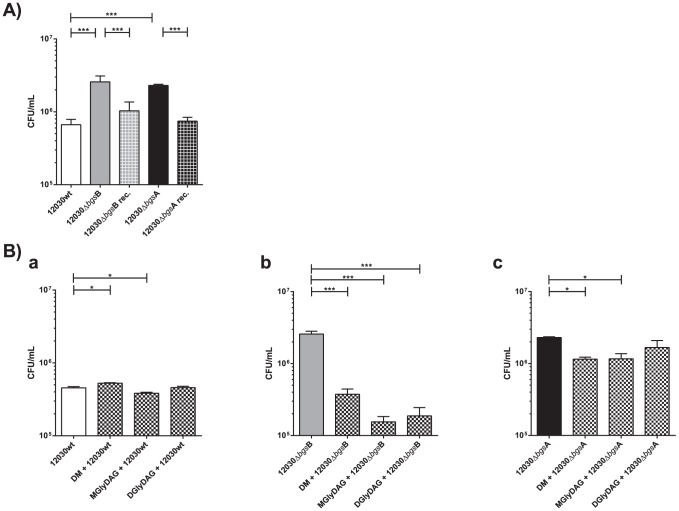
Attachment to T24 cells. (A) *E. faecalis* 12030 wild-type (white bar), *E. faecalis* 12030Δ*bgs*B mutant (grey bar), reconstituted *E. faecalis* 12030Δ*bgs*B mutant (grey squared bar), *E. faecalis* 12030Δ*bgs*A mutant (black bar) and reconstituted *E. faecalis* 12030Δ*bgs*A mutant (black squared bar) were tested for their ability to bind to T24 cells. T24 cells were incubated for 2 hours with bacteria grown to mid-log phase at a bacteria-to-cell ratio of 100∶1. Total cell-associated bacteria include surface-adherent and intracellular bacteria. Both mutants showed clearly increased binding compared to the wild-type strain and the reconstituted mutant showed reduce binding compared to the mutants. Data represent the means with standard error of the mean (SEM). B) Inhibition of bacterial attachment to T24 cells using glycolipids. *E. faecalis* 12030 wild-type (a, white bar), *E. faecalis* 12030Δ*bgs*B mutant (b, grey bar) and *E. faecalis* 12030Δ*bgs*A mutant (c, black bar) were tested. T24 cells were incubated with glycolipids (M: MGlyDAG, D: DGlyDAG and DM: mixture of MGlyDAG and DGlyDAG) for 30 min before addition of bacteria to assess attachment. Each glycolipid alone and the mixture of both significantly reduced bacterial binding using ANOVA with Bonferroni's multiple comparison test. For all inhibitions using glycolipids, differences were statistically significant at p<0.001. Bars represent average ± S.E.

*E. faecalis* 12030Δ*bgs*B and *E. faecalis* 12030Δ*bgs*A mutants were investigated for their binding to T24 bladder carcinoma cell line. These mutants showed a more than three-fold increased binding (2.6×10^6^ CFU/mL and 2.3×10^6^ CFU/mL) to the T24 human bladder carcinoma cell line compared to wild-type bacteria (0.7×10^6^ CFU/mL, Figure 1A). The increased attachment was significantly lower (more than 50%) with the complemented strains of both mutants *E. faecalis* 12030Δ*bgsB* rec and *E. faecalis* 12030Δ*bgsA* rec (1.1×10^6^ CFU/mL and 0.8×10^6^ CFU/mL, Figure 1A).

### Bacterial attachment is inhibited by purified glycolipids

To study the specific interaction between bacteria and a still-unknown host receptor, T24 cells were first incubated with purified glycolipids DGlcDAG, MGlcDAG, or a mixture of both to saturate binding sites before inoculation with bacteria. As shown in figure 1B, the *E. faecalis* 12030Δ*bgs*B mutant showed significantly reduced binding up to 94% to the bladder epithelial cell line after pre-treatment with glycolipids. Inhibition of each of the purified lipids was higher than inhibition by a mixture of them. The inhibition of binding using mixture of both glycolipids was 87% (Figure 1B, b). The binding of *E. faecalis* 12030Δ*bgs*A mutant was significantly reduced up to 50% using glycolipids (Figure 1B, c). However, no inhibition effect was seen in the wild-type strain (Figure 1B, a).

### LTA and WTA are not involved in *E. faecalis* binding to bladder epithelial cell line T24

**Figure 2:**
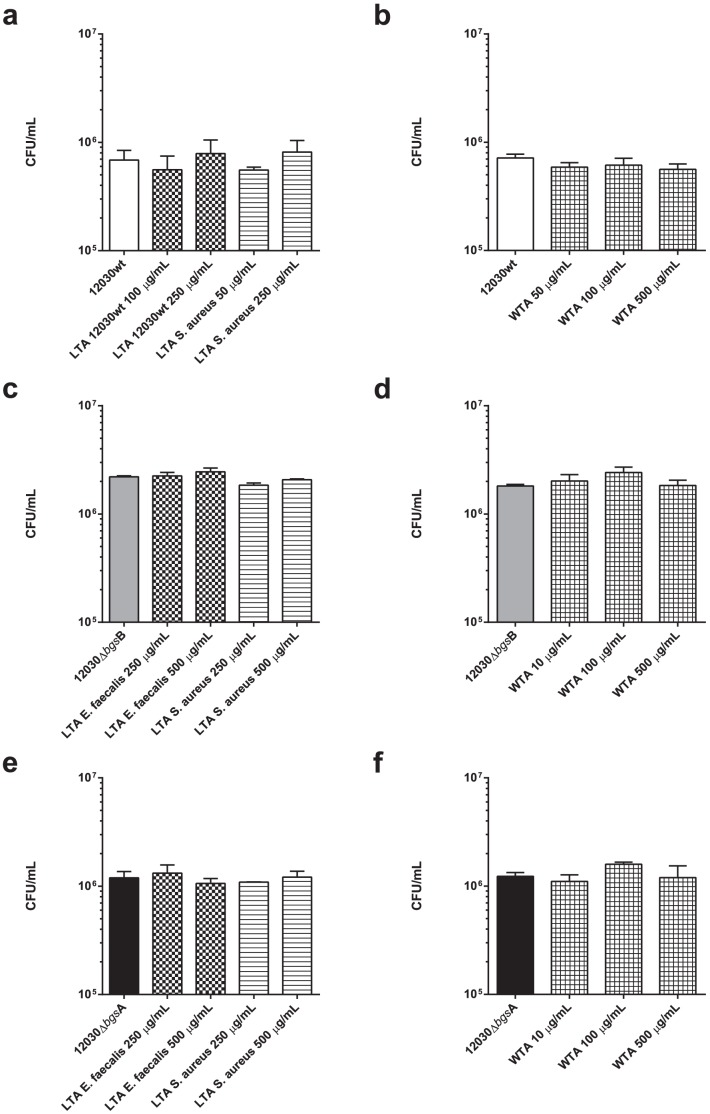
Inhibition of bacterial binding to T24 epithelial cells using WTA and LTA. Epithelial cells were incubated with different concentrations of WTA (A) and LTA (B) 30 min before addition of *E. faecalis* strains. *E. faecalis* 12030 wild-type (white bar), *E. faecalis* 12030Δ*bgs*B mutant (grey bar), *E. faecalis* 12030Δ*bgs*A mutant (black bar). Concentrations of WTA were between 50 and 1000 µg/mL, and concentrations of LTA were between 100 and 250 µg/mL for the prepared LTA and between 50 and 250 µg/mL for the LTA purchased from Sigma. In both cases, bacterial binding to T24 cells was not significantly impaired when using LTA and WTA. Using ANOVA test the difference was statistically not significant (p>0.05). Bars represent average ± S.E.

Two different LTA preparations were used for inhibition of binding to T24 cells: one purchased from Sigma (LTA from *S. aureus*), another prepared from *E. faecalis* strain 12030 according to the method described by Morath et al. [19], (100 and 250 µg/mL). We used LTA from *S. aureus* to test if this leads to different results compared to *E. faecalis* LTA, because both LTAs are different in structure, as described by Weidenmaier and Theilacker [20], [21]. In the LTA of *E. faecalis*, the C-2 hydroxyl is glycosylated either by kojibiose (α-D-glucopyranosyl-(1→2)-α-D-glucose) or 6, 6′-di-alanyl-α-kojibiose [21]. LTA from *Staphylococcus aureus* is partially substituted at the C-2 hydroxyl with α-D-GlcNAc [20]. Wall teichoic acid (WTA) was prepared as described elsewhere and used at (10–500 µg/mL). Wild-type strain *E. faecalis* 12030 and both glycolipid mutants were tested. As shown in figure 2, none of these compounds had a significant effect on bacterial binding of these strains.

### Glycosaminoglycans are not involved in *E. faecalis* attachment to bladder cell line T24

**Figure 3:**
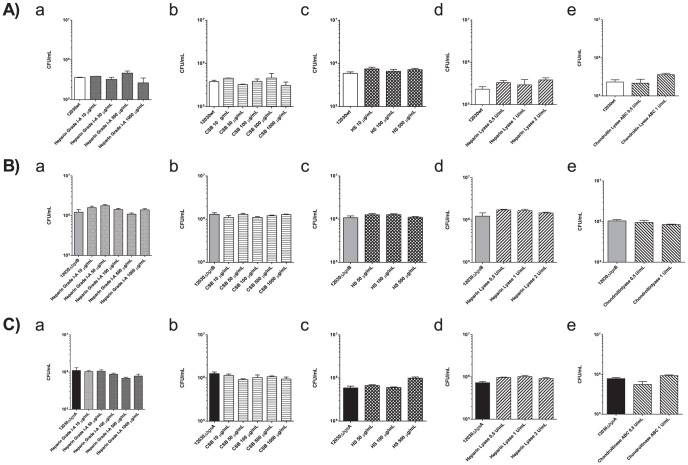
Effect of different glycosaminoglycans and their lyases on attachment of *E. faecalis* 12030 strains to T24 uroepithelial cells. *E. faecalis* 12030 wild-type (A, white bar), *E. faecalis* 12030Δ*bgs*B mutant (B, grey bar) and *E*. *faecalis* 12030Δ*bgs*A mutant (C, black bar) were used. Binding of this strains to the confluent monolayers of uroepithelial cells was investigated in the presence of H/heparin (a, 10–1000 µg/mL concentrations), CSB/chondroitin sulfate (b, 10–1000 µg/mL concentrations), HS/heparin sulfate (c, 10–500 µg/mL concentrations), heparin and chondroitin lyase ABC (d, e, heparin lyase 0.5–2 units/mL and chondroitin lyase ABC 0.5–1 units/mL) were used to digest the GAGs chains for 10 min prior to and 2 h after bacterial infection. In all cases an inhibition of bacterial binding to T24 cells were not possible. Bars represent average ± S.E. Using ANOVA test were no significant differences for all (p>0.05) data.

Previous results demonstrated a role of glycosaminoglycans (GAGs) in adhesion to gastrointestinal epithelial cells [22], [23]. To investigate the role of GAGs in *E. faecalis* binding to bladder epithelial cells, we used heparin, heparan sulfate, and chondroitin sulfate. As shown in figure 3, no significant inhibition of bacterial binding to T24 cells was observed with any of the GAGs using the wild-type strain (Figure 3 A, a–c) and both glycolipid mutants (Figure 3B and 3C, a–c). Although we did not see an effect with different GAGs, we performed additional experiments using enzymatic digestion of GAGs on target cells. As expected, digestion of GAGs with heparin lyase (Figure 3d) and chondroitin lyase (Figure 3e) did not yield any significant change in binding for all strains.

### Proteinase K and sodium meta-periodate have no effect on *E. faecalis* binding to uroepithelia

**Figure 4:**
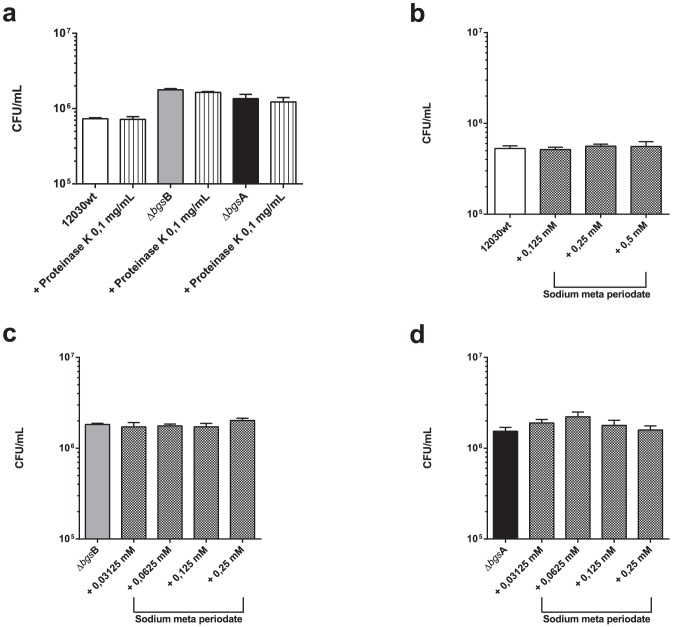
Effect of Proteinase K and sodium meta-periodate treatment of *E. faecalis* glycolipid mutants and wild-type strain on attachment to T24 uroepithelial cells. The mutants *E. faecalis* 12030Δ*bgs*B, *E. faecalis* 12030Δ *bgs*A and wild-type strain 12030 were treated with Proteinase K (a, 0.1 mg/mL) and sodium meta periodate (b–d, 0.03125–0.5 mM). Bacteria grown in the same conditions without the proteinase K and sodium-meta periodate were used as controls. There were no significant differences (p>0.05) calculated with ANOVA. Bars represent average ± S.E.

To determine whether bacterial surface proteins are involved in the binding of mutants to bladder cells, we treated both mutant strains and the wild-type strain with proteinase K (0.1 mg/mL) prior to attachment. After proteolytic treatment neither the glycolipid mutant nor the wild-type strain showed any significant decrease in binding to T24 cells (Figure 4a). Sodium meta-periodate was used to assess the role of bacterial polysaccharides in adhesion. Both glycolipid mutants and wild-type strain were treated with different concentrations of sodium meta-periodate (0.03–0.5 mM) before they were incubated with T24 cells, and no obvious difference in bacterial binding was detected (Figure 4b–d).

### *E. faecalis* glycolipid mutants colonize uro-epithelial surfaces more efficiently than wild-type *E. faecalis*

**Figure 5:**
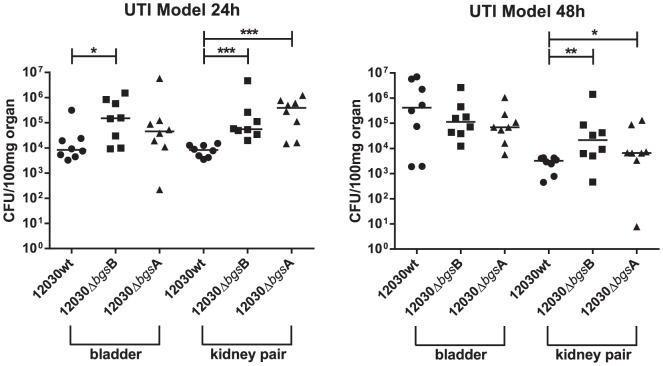
Monoinfection with wild-type *E. faecalis* 12030 (12030wt) and its glycolipid mutants *E. faecalis* 12030Δ*bgs*B and *E. faecalis* 12030Δ*bgs*A. Data are from 8 mice infected with 10^8^ CFU; results are expressed as log10 CFU per 100 mg from kidney and bladder homogenates 24 h and 48 h after transurethral challenge. The log10 CFUs from both kidneys were combined and averaged. A value of 1 was assigned to those kidneys with 0 CFU. Circles represent wild-type *E. faecalis* 12030, squares represent *E. faecalis* 12030Δ*bgs*B, triangles represent *E. faecalis* 12030Δ*bgs*A. Horizontal bars represent the geometric mean. The mean difference in CFU counts of mutants versus 12030 is given as the log10± the standard deviation (SD). Differences in the log10 CFU of *E. faecalis* 12030 wild type versus the *E. faecalis* 12030Δ*bgs*A and 12030Δ*bgs*B mutants are given.

To study the role of glycolipids in the pathogenesis of urinary infection, a modified mouse urinary infection model was used. Female BALB/c mice 6–8 weeks old were inoculated with 100 µl 10^8^ CFU of bacteria transurethrally. The animals were re-inoculated two hours later with the same amount of bacteria to achieve consistent infection. The bacterial counts in the bladder and in both kidneys were determined 24 and 48 hours post-infection as described elsewhere [9]. Both glycolipid mutants colonize the kidneys and bladder significantly better than the wild-type strain after 24 hours. Concentrations of the *E. faecalis* 12030Δ*bgs*B mutant was 1.2×10^5^ CFU and the wild-type strain was 3.9×10^3^ CFU in the bladder. Both mutants *E. faecalis* 12030Δ*bgs*B and *E. faecalis* 12030Δ*bgs*A colonized the kidneys up to 8×10^4^ CFU and 3×10^5^ CFU and for the wild-type strain was 8×10^3^ CFU after 24 hours. After 48 hours the wild-type strain colonized the bladder 1.5 log10 CFU more compared to 24 hours while both mutants showed no significant differences at this time point. Both mutants colonized the kidneys significantly better after 48 hours (Figure 5).

## Discussion

Tight adhesion of microorganisms to mucosal surfaces of the urinary tract is important for the pathogenesis of UTIs, because the mechanical removal of colonizing bacteria by the urine flow is an important innate defence mechanism. In the process of bacterial adherence, infectious agents interact with specific molecules on epithelial cells [24], [25]. It has been demonstrated that the tendency of certain bacteria to infect specific tissues is related to their ability to adhere to specific target cells [26]. The P fimbriae of the urinary pathogen *Escherichia coli* (UPEC) recognize as receptors both Galactose α 1-4Galactose β disaccharide (Galα 1-4Galβ) and N-acetyl-D-galactose amine (GalNAc(β1-3)Gal(α1-4)Galβ), which contain oligosaccharide sequences in the globoseries of glycolipids [27], [28]. Several virulence determinants such as cytolysin, gelatinase, extracellular superoxide, surface-exposed proteins (e.g., Ace, EfaA, Esp, AS, epa), and surface carbohydrates of *E. faecalis* have been studied previously [29], [30], [31], [32], [33]. However, no study has focused on the possible role of glycolipids in UTI infection with gram-positive bacteria such *E. faecalis*.

We therefore studied the previously described *E. faecalis* 12030Δ*bgs*B and *E. faecalis* 12030Δ*bgs*A mutants with regard to binding to T24 bladder carcinoma cells and colonization of the urinary tract in mice. We showed previously that these mutants do not bind to Caco2 colonic epithelial cells equally well as the wild-type strain [13], [14]. However, here we observed that both mutants showed a more than three-fold increased binding to human bladder carcinoma cells compared to the wild-type strain. We hypothesize, that this may be depend on the so-far unknown surface receptors of both epithelial cells and also on growth-conditions, such as pH and salt concentration. Moreover, to examine the effects of the specific interaction between glycolipids on attachment of *E. faecalis* and a still-unknown host receptor, T24 cells were first incubated with purified glycolipids DGlcDAG, MGlcDAG, and a mixture of both glycolipids of *E. faecalis* 12030 strains to saturate specific binding sites of the putative host receptor for glycolipids. In contrast to the wild-type strain, the binding of both *E. faecalis* glycolipid mutants to human bladder cells was significantly reduced and was also dose-dependent (data not shown). Interestingly, *E. faecalis* 12030Δ*bgs*B mutant missing both glycolipids showed a stronger reduction in binding (up to 95%) as the *E. faecalis* 12030Δ*bgs*A mutant (up to 50%), which is missing only DGlcDAG. This confirms the specificity of binding and suggests the existence of specific receptors for glycolipids in the host. However, a mixture of both glycolipids inhibited binding to a lesser extent than the single purified glycolipids which may be explained by the higher concentration of the respective glycolipids in the more purified preparation.

To assess the relevance of these results *in vivo*, a modified mouse urinary infection model was used. The results confirmed that both glycolipid mutants (i.e. *E. faecalis* 12030Δ*bgs*B and *E. faecalis* 12030Δ*bgs*A) colonize the kidneys at 48 hours significantly better than the wild-type strain. Also, both glycolipid mutants colonize the bladder significantly better than the wild-type strain after 24 hours. However, colony counts after 48 hours showed that long-term colonization of the bladder was not significantly different between wild-type and mutants. Interestingly, the wild-type strain colonized the bladder better after 48 hours compared to 24 hours. However both mutants showed similar colonization. Figure 5 clearly indicates that the wild-type strain and both mutants behave differently in the bladder. *E. faecalis* wild-type persists significantly better in the bladder after several hours.

This may explain the fact, that in contrast to glycolipid mutants, *E. faecalis* wild-type could induce additional factors to persist in the bladder. The absence of glycolipids seems to favour the adherence of bacteria to uroepithelia, which is in contrast to our previous results with a mouse sepsis model [13], [14]. Early work in this field showed that a specific glycolipid, alpha-galactosylceramide (GalCer), had an inhibitory effect in a mouse urinary tract infection model using wild-type *E. coli* [34]. Together with our results, this indicates the presence of specific receptors for glycolipids on bladder/uroepithelium cell surfaces.

The loss of both glycolipids (i.e., monoglucosyl-diacylglycerol and diglucosyl-diacylglycerol; [13], [14]) may induce compensatory changes in the concentration of other polar lipids of the cell membrane, although we previously showed that total phospholipids and aminophosholipids did not differ significantly among the two mutants and wild-type bacteria [13]. Our previous results indicated that the deletion of the glycosyltransferases *bgsA* and *bgsB* also affected the synthesis of LTA [13], [14]. This, together with studies demonstrating that LTA is involved in adherence to epithelial surfaces [35], [36], [37], indicates a possible role of the modified LTA in adherence to urinary epithelia. LTA produced by the glycolipid mutants is larger and contains more kojibiose and alanine substituents at the C2 of glycerol [13]. Hence, modifications of the LTA and possibly also compensatory WTA modifications could be responsible for the increased adherence of the glycolipid mutants to bladder and kidney cells. With our findings of inhibition of binding by externally applied glycolipids, these results point toward a possible role for inhibition of bacterial adherence to bladder and kidney cells through LTA or glycolipids. These findings corroborate our previous work showing the inhibition of binding of enterococci to colonic Caco2 cells with glycolipids [18].

Several MSCRAMM surface proteins have been implicated in the adherence of enterococci to host cells [32], [38], [39], [40], [41], [42]. To investigate whether such proteins are involved in the adherence of mutants to bladder cells, we treated both mutants and the wild-type strain with proteinase K. After enzymatic treatment there were no significant differences in adherence, indicating no or only a minor role for proteins in bacterial adhesions. However it is well known that the majority of LPXTG surface proteins (pilins, some MSCRAMMs) are protease resistant due to intramolecular amide bonds. Also, a dose-dependent treatment of bacteria with sodium meta-periodate showed no differences in bacterial binding of both mutants and the wild-type bacteria. This indicates that a carbohydrate moiety is probably not responsible for the greater adherence of both glycolipid mutants to uroepithelial T24 cells. In contrast, a human colon carcinoma cell, Caco2 and *E. faecalis* wild-type bacteria, showed a decrease of adherence after sodium meta periodate treatment indicating that a carbohydrate moiety is responsible for binding in this setting [13].

There is little information on glycolipid receptors in gram-positive uropathogenic bacteria. However, glycosaminoglycans (GAGs) on the surface of mammalian epithelial cells function often as receptors for human pathogens [22], [23]. In our previous experiments we demonstrated that adherence of a *E. faecalis* wild-type strain to human Caco2 colon carcinoma cells was mediated by interactions between bacterial glycolipids and highly sulfated glycosaminoglycans (GAGs) such as heparin on the surface of carcinoma cells [18]. This interaction was inhibited by the purified glycolipid DGlcDAGs of *E. faecalis*, but not by LTA or WTA [18].

Based on these findings we examined the possibility that GAGs act as receptors for enterococcal adherence of glycolipids to uroepithelia. In contrast to our previous results, we found that heparin, HS, and CSB are not primarily involved in adherence of wild-type bacteria and glycolipid mutants to uroepithelial cells. We were not able to inhibit binding using purified GAGs, and saw no difference after digestion of GAGs on the uroepithelial cell surface with enzymes such as heparin lyase and chondroitin lyase ABC.

In summary, we demonstrated that the deletion of *bgs*B and *bgs*A leads to increased binding of uroepithelial cells and greater bladder colonization in a mouse UTI model. Inactivation of both enzymes leads to a depletion of the major glycolipid, diglucosyl-diacylglycerol, which is involved in adhesion and biofilm formation [13], [14]. We hypothesize that diglucosyl-diacylglycerol is released into the culture supernatant and blocks host binding sites (receptors), thereby preventing bacterial adhesion. Since diglucosyl-diacylglycerol is missing in both the *bgsA* and *bgsB* mutant, bacteria can probably interact better with the host epithelia through their (modified) LTA or WTA. The release of glycolipids into the culture supernatant of the wild type, but not the mutants, was confirmed by thin-layer chromatography [14]. Further experiments will be needed to clarify the exact mechanism of the adhesion through glycolipids and their cognate receptors.
